# Interventions to improve social support among postpartum mothers: A systematic review

**DOI:** 10.34172/hpp.2022.18

**Published:** 2022-08-20

**Authors:** Foruzan Sharifipour, Mojgan Javadnoori, Zahra Behboodi Moghadam, Mahin Najafian, Bahman Cheraghian, Zahra Abbaspoor

**Affiliations:** ^1^Reproductive Health Promotion Research Center, Department of Midwifery, Faculty of Nursing and Midwifery, Ahvaz Jundishapur University of Medical Sciences, Ahvaz, Iran; ^2^Reproductive Health Promotion Research Center, Department of Midwifery, Ahvaz Jundishapur University of Medical Sciences, Ahvaz, Iran; ^3^School of Nursing & Midwifery, Tehran University of Medical Sciences, Tehran, Iran; ^4^Department of Obstetrics and Gynecology, School of Medicine, Fertility Infertility and Perinatology Research Center, Ahvaz Jundishapur University of Medical Sciences, Ahvaz, Iran; ^5^Alimentary Tract Research Center, Clinical Sciences Research Institute, Department of Biostatistics and Epidemiology, school of Public Health, Ahvaz Jundishapur University of Medical Sciences, Ahvaz, Iran

**Keywords:** Intervention education, Social support, Postpartum period, Systematic review

## Abstract

**Background:** Poor and insufficient social support to the mother in the post-partum period impairs the effective functioning of her new role as a mother, and it is an important risk factor for the maternal depression and stress after childbirth. Thus, interventions to improve social support to mothers in their postpartum period are required. The present review aimed to investigate the effectiveness of the existing interventions aimed at improving social support among postpartum women.

**Methods:** In this systematic review, PubMed, Scopus, Science Direct, Cochrane Library, Web of Science, EMBASE, Google Scholar, IranDoc, IranMedex, MagIran and SID were searched until January 2022. Full-text articles on the social support outcome, published in English or Persian, which used the design of randomized controlled trials (RCTs) or comparison groups and involved postpartum or pregnant women as participants were included. The quality of the studies was assessed based on the seven criteria offered by Cochrane guidelines.

**Results:** Our review included 10 studies involving 3328 women. According to our results, the following interventions were successful in increasing social support compared to the controlled conditions: counseling with men in the prenatal period, interventions based on interpersonal psychotherapy (IPT), advanced practice nurse (APN), internet support, and home visiting in the postpartum period.

**Conclusion:** These interventions could be provided to mothers during their prenatal or postpartum care. However, which one of these interventions is the most effective in improving social support among postpartum mothers was not identified in the present study.

## Introduction

 Pregnancy and delivery along with the postpartum period represent very important milestones in women’s life, which not only affect their lives, their children and husbands, and their relatives, but also exert a profound effect on society as a whole.^1^ Nearly 20%-40% of postpartum mothers suffer from mood disorders that prevent them from caring for their newborns.^2^ During this period, the mother is worried about her and her newborn’s health and experiences changes in her physical, psychological, sexual, and social status. These changes can lead to postpartum stress and impair effective functioning in her new role as a mother.^3^ Therefore, helping mothers and promoting their health is of paramount importance in this period. For example, undesirable consequences could be prevented by support programs and proper caring for mothers and infants.^4^ Most primiparous mothers are unprepared to become mothers and need support from family, friends, and healthcare providers.^5^ In this respect, social support plays a very important role in reducing these mothers’ vulnerability and stress.^6^

 Social support is the respect, sense of belonging, love, and affection that a person receives from others.^7^ According to a meta-analysis by Ghasemi et al, providing social support is one way to promote a healthy lifestyle among women.^8^ Social support has three main types, namely (1) Emotional social support: having someone available to rely on and to trust when needed (2), Instrumental social support: the material, objective and real assistance received by an individual from others, and (3) Information social support: obtaining essential information through social interactions with others.^9^ Individuals receiving social support enjoy several psychological benefits: their self-confidence is promoted, they feel empowered and efficient, and their quality of life is improved.^10-12^ Studies have shown that poor social support or lack of it is an important risk factor for depression and stress in the postpartum period.^13-18^ Women in the postpartum period not only must get rid of the problems caused by the delivery process and take care of their newborn, but also need social support to help them cope with these stresses.^19^

 Insufficient social support for mothers in the postpartum period has adverse consequences. The literature reports no systematic review to examine the interventions performed to improve social support for these mothers. Therefore, this study aimed to examine and provide a summary of the current literature on interventions used to improve social support for mothers in the postpartum period.

## Materials and Methods

###  Search strategy

 This systematic review was carried out according to the PRISMA checklist.^20^ The databases that were searched included PubMed, Web of Science, Scopus, Cochrane Library, Science Direct, EMBASE, Google Scholar, IranDoc, IranMedex, MagIran and SID. The initial search lasted until August 2020, and it was later updated until January 2022. The combination of terms searched in this study included: “Social support” OR “social care” OR “social aid” OR “public assistance” OR “social assistance” AND “intervention” OR “multidisciplinary intervention” ،OR “program” OR “plan” OR “package” AND “postpartum period” OR “postpartum care” OR “postpartum women” OR “postpartum Program” OR “postnatal period” OR “postnatal care” OR “puerperium” OR “puerperium care”.

###  Study selection

 Studies were eligible to be included in this review if they: (1) investigated the interventions dealing with social support of postpartum mothers; (2) included postpartum mothers or pregnant women as participants; (3) were published full-text in English or Persian; and (4) had the design of randomized controlled trial (RCT) or quasi experimental. Studies with no full texts or irrelevant results, as well as duplicate studies and animal experiments were excluded from the study. FS and ZA screened the titles and abstracts of all studies obtained according to the inclusion criteria. The full texts of the studies meeting the inclusion criteria were thoroughly examined. Finally, those papers dealing with the outcomes of interventions aimed at maternal social support in the postpartum period were selected. Screening process was done using Covidence website which made removal of duplicate articles possible. The authors resolved possible disagreements by discussion.

###  Quality assessment of the included studies

 The risk of bias for each study was evaluated using the seven criteria offered by Cochrane guidelines for assessing the quality of RCTs. These seven criteria concluded: (1) random sequence generation (selection bias), (2) allocation concealment (selection bias), (3) blinding of the participants and the personnel (performance bias), (4) blinding of outcome assessment (detection bias), (5) incomplete outcome data (attrition bias), (6) selective reporting (reporting bias), and 7- other risks of bias (including sample size calculation, study power, obtaining consent from participants, and approval of the ethics committee). There are three options for each criterion: low risk, unclear and high risk.^21^ Two independent reviewers (FS and ZA) assessed the risk of bias of the included studies.

###  Data extraction

 FS independently extracted the data from the final studies while ZA and ZB checked the data for precision and completeness. Authors negotiated any conflict to achieve agreement. The variables of interest were extracted from the selected studies. These included the first author’s name, date of publication, place of research implementation, design, intervention type, size of the sample, characteristics of the participants, outcomes, tools, and findings.

## Results

###  Search results

**Figure 1:**
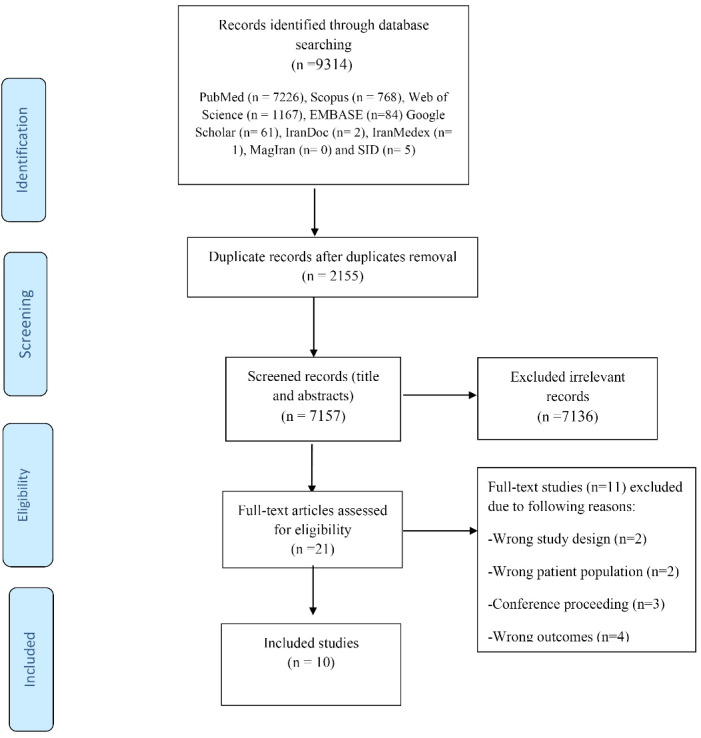
Flowchart of the process of searching and selecting studies

 The selection of studies in this review is depicted in the PRISMA chart (Figure 1).

 The initial search of the databases involved 9314 studies of which 2155 were removed due to being duplicates. The remaining 7157 articles were screened according to their titles and abstracts, which resulted in the exclusion of 7136 irrelevant articles. There left only 21 articles the full text of which was screened. This led to the exclusion of two studies due to wrong study design, two studies due to wrong patient population, four studies due to imprecise outcomes, and three studies due to being included in conference proceedings. Eventually, 10 full-text articles were selected as the final articles to be analyzed in this systematic review.

###  Quality of the included studies

**Figure 2:**
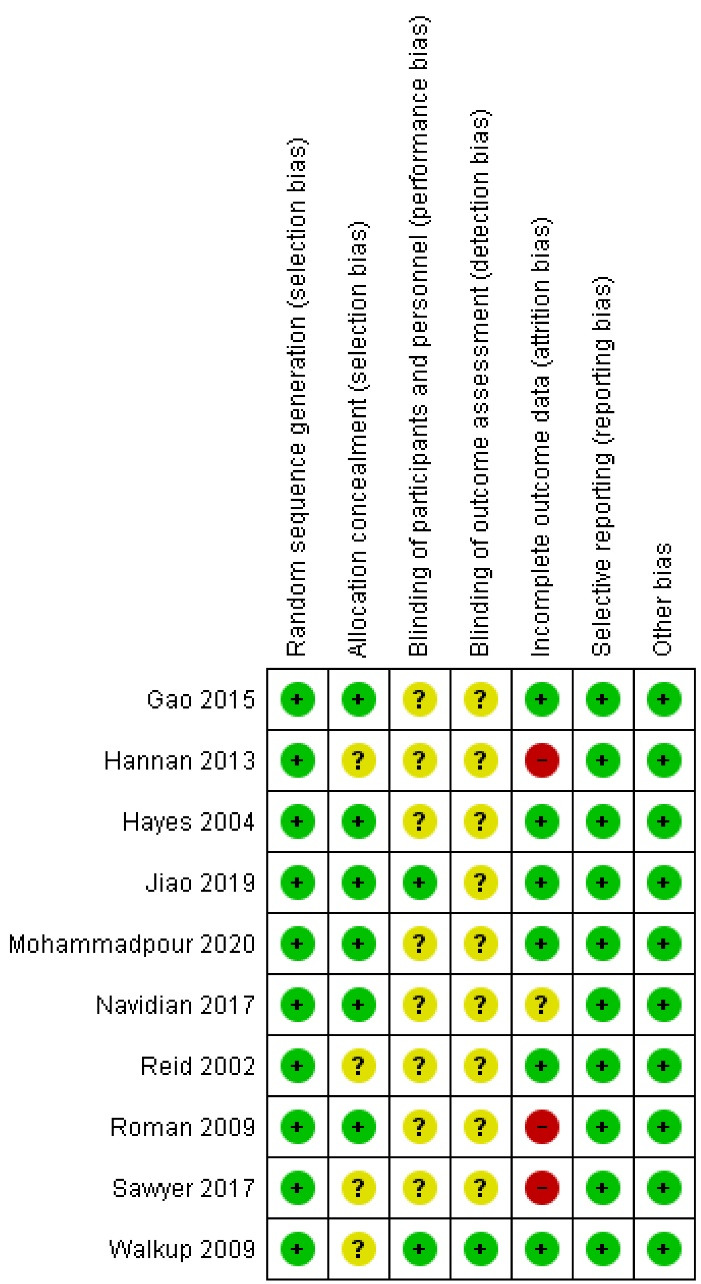
Quality assessment for each included study assessed with 7-item list

**Figure 3:**
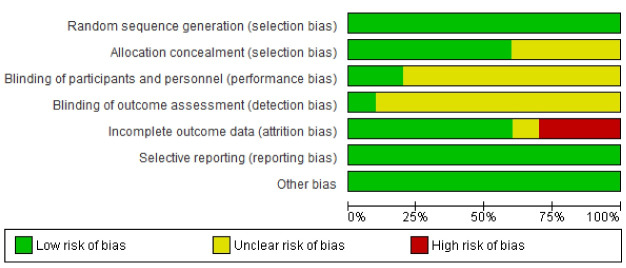
Quality assessment about each risk of bias item presented as percentages across all included studies

 All articles were within the low-risk range in terms of random sequence generation, selective reporting, and other types of bias. For allocation concealment, six out of the ten studies had low risk bias, and the other four studies reported no information regarding allocation concealment; thus they had unclear risk. As far as blinding of the participants and the personnel was concerned, 2 studies were rated as low risk, and the other 8 studies reported no information on this factor because the nature of the interventions in these studies did not allow blinding of the participants and the personnel. With respect to attrition bias, 6 articles were considered as low risk (Figure 2). Figure 3 shows the risk of bias appraisal in percentages.

###  Description of the studies

Table 1 summarizes the main characteristics of the studies included in this review. The publication date of the selected articles ranged from 2002 to 2020. In terms of place of research implementation, five were in Asia,^22-26^ one in Europe,^27^ two in the USA,^28,29^ and two in Australia.^21,22^ As far as the study design was concerned, seven were RCTs,^22-24,26,28,29^ one was quasi-experimental,^25^ one was RCT with a 2 × 2 factorial,^27^ and one was randomized pragmatic non-inferiority.^30^ Overall, the participants of this review included 2446 primiparous mothers giving birth to single full-term healthy babies, and 882 expecting mothers at 20–24 weeks of gestation, with singleton pregnancy and first gravidity.

 In three studies, interventions were performed during the prenatal period^24,29,31^ and included the following four interventions: (1) counseling with men (2) nurse-community health worker (CHW) (3) home visiting, and (4) educational package. In the other seven studies, interventions were performed in the postpartum period^22,23,25-28,30^ and included the following six interventions: (1) interpersonal psychotherapy (IPT), (2) advanced practice nurse (APN), (3) internet-based support, (4) Home visiting, (5) self-help manual, and (6) support group.

 Any intervention performed in studies during the prenatal and postpartum periods is described in more details as follows.

**Table 1:** Characteristic of the included studies

Author and year	**Country**	**Study design**	**Sample size**	**Participant characteristics**	**Intervention**	**Control**	**Follow up**	**Outcome**	**Findings**
Prenatal period									
Roman^29^	USA	RCT	N=613	pregnant women at <24 weeks gestation	Nurse-CHW	Community care	15 months post birth	MSPSS	No differences between the groups were found social support.
Mohammadpour et al^24^	Iran	RCT	n= 102	Pregnant women with gestational age of 20–24 weeks, singleton pregnancy and first pregnancy who their spouses were willing to attend counselling classes	Counselling with men	Routine care	4 weeks after intervention	Social support PRQ-85-Part 2	Four weeks following the intervention, the social support mean score rose significantly in the counseling group in comparison to control group.
Hayes and Muller^31^	Australia	RCT	N=184	Pregnant women at >12 weeks gestation	Education package	Routine care	8-12 weeks postpartum and 16-24 weeks postpartum	NSSQ	There were no significant differences in social support score between both groups at all post-tests.
Postpartum period									
Gao et al^22^	China	RCT	n=180	First-time mothers delivering single full-term health infant	IPT	Routine postnatal care	6 weeks postpartum	PSSS	There was a higher mean score of social support in intervention group.
Hannan^28^	USA	RCT	n= 139	First-time low-income mothers, in good health, who gave birth to a singleton healthy full term baby	APN	Routine care after discharge from hospital	Day 3, months 1 and 2	MSPSS	Compared with the control group, mothers in the intervention group had a superior perception regarding social support at post hospital discharge month 2.
Jiao et al^23^	Singapore	RCT	n= 204	First-time mothers giving birth to a singleton healthy baby	Web based group, Homebased group	Routine care	1, 3, and 6 months after delivery	PICSS	Compared with the control group, mothers in both web-based and home-based interventions had better social support scores at all post-tests.
Navidian et al^25^	Iran	quasi-experimental	N=100	First-time mothers who had given birth with single full-term health baby and routinely referred to health centers for receiving postpartum care on the third to fifth day after delivery	Home-based supportive educational counseling	Usual postpartum care	The end of sixth post-delivery week	HPSS	Compared to the control group, the intervention group received significantly lower scores in terms of lack of social support after a home-based supportive-educational intervention.
Reid et al^27^	Scotland	RCT with a 2 × 2 factorial	N=1004	Primiparous women	- A postnatal support manual (‘pack’) - A invitation to attend a support group (‘group’)	Routine care	3 and 6 months after delivery	SSQ6	No significant differences were found between the control and trial groups in terms of SSQ6 scores 3 and 6 months after delivery.
Sawyer et al^30^	Australia	pragmatic, preference, randomized, non-inferiority	N=819	New mothers who referred to clinics for their initial postnatal health check	Internet-based support, home-based support	-	9, 15 and 21 months post birth	MSS	Differences did not exceed the pre-specified margin of inferiority in outcomes between mothers in the Internet-based and home-based support groups.
Walkup et al^26^	Indian	RCT	n = 167	Pregnant women at 28 weeks or lesser of gestation	Home visiting interventions	Breast-feeding/nutrition education intervention	2, 6, and 12 months after delivery	Social support CESD	No between-group differences were found for mothers social Support.

IPT: Interpersonal psychotherapy, APN: Advanced practice Nurse, PSSS: Perceived Social Support Scale, MSPSS: Multidimensional Scale of Perceived Social Support, PICSS: Perinatal Infant Care Social Support, PRQ-85-Part 2: Personal Resource Questionnaire-85-Part 2, HPSS: Hung Postpartum Stress Scale, SSQ6: Social Support Questionnaire- Short Form, MSS: Maternal Support Scale, CESD: Center for Epidemiological Studies – Depression, NSSQ: Norbeck Social Support Questionnaire; CHW, community health worker.

###  Interventions in the prenatal period and their length

####  Counseling with men

 The intervention by Mohammadpour et al involved four sessions of counseling for husbands of pregnant women at 20–24 weeks of gestation, and the length of each session was approximately 1 hour. For four consecutive weeks, counseling sessions were held once a week. The outline of the counseling sessions included the effect of social support on the mother and fetus during pregnancy, the role of fathers in supporting pregnant women, the effects of not supporting pregnant women and its outcomes after childbirth, the importance of fathers’ relationship with the fetus and their duties during pregnancy and childbirth, sexual relations and their changes in pregnancy, mental health during pregnancy, anatomical, physiological, and hormonal changes during pregnancy, the effect of such changes on the body especially the mother’s mental state, providing solutions to better cope with these changes, fathers’ role in helping mothers to adapt to these changes, and the stages of fetus development during pregnancy. Routine care was provided to the control group. Prior to intervention and 4 weeks after it, effects were assessed. The results indicated that in comparison with the control group, there was a significant increase in the mean score of social support in the counseling group 4 weeks after intervention (MD: 12.7; 95% CI: 18.5 to 6.9).^24^

####  Community health worker 

 Roman et al used CHW intervention involving a nurse along with two CHWs functioning as a team. Women having a high school diploma were recruited as CHWs. Monthly CHW educational sessions included relationship building, problem solving, goal setting, stress management, self-esteem, and assertiveness. Nurses were required to make at least two prenatal visits, one postpartum visit, and two extra visits within the postpartum year whenever possible. However, they could make more visits depending on the health of the mother and baby. CHWs provided support on telephone and through face-to-face encounters. The control group received community care. Measurement of outcomes was done at baseline (prenatal) and 15 months after childbirth. According to the results of their study, no difference was observed between social support scores of the two groups.^29^

###  Home visiting

 In Walkup et al, home-visiting interventions included 25-visit ‘‘Family Spirit’’ intervention which dealt with prenatal and newborn care as well as life skills for pregnant women with 28 weeks or shorter gestation period. The interventions, which were offered by well-trained paraprofessionals, were carried out at the participants’ homes or in a private environment at the participants’ convenience. The lesson content of the Family Spirit intervention was according to the American Academy of Pediatrics’ Caring for Your Baby and Child: Birth to Age 5.^32^ The program involves lessons on prenatal and baby-care, substance abuse prevention, problem solving, coping-skills, and family planning. The intervention of control group included 23-visit lessons on breast-feeding/nutrition. The interventions started during pregnancy and lasted at most for 6 months after delivery. Outcomes were measured at baseline and 2, 6, and 12 months postpartum. According to the results, no significant difference was found between groups in terms of mothers’ social support at the three mentioned intervals.^26^The length of each home visit was not mentioned.

###  Educational package

 Hayes and Muller used an educational package as their intervention. The package included: (1) an information booklet devoted to expecting mothers, their husbands and extended families; (2) an audiotape of a woman suffering from post-natal clinical depression who recovers from it after a while; and (3) an experienced midwife to guide the mothers. It involved six information categories: (1) Pregnant women’s history of mood changes; (2) Information about the cause of mood changes; (3) Information about the women’s different symptoms; (4) Information on seeking assistance (time, place, and quality); (5) Information on the development of a personal plan for seeking and receiving help; (6) Information for partners, extended family members, and friends. Only routine care was provided to the control group in this study. Outcome measurements were at 12-28 weeks of gestation, along with 8-12 and 16-24 weeks after delivery. According to the results of this study, no significant difference was reported between the two groups in terms of social support.^31^

###  Interventions in the postpartum period and their length

####  Interpersonal psychotherapy

 Gao and colleagues’ study involved an interpersonal-psychotherapy-oriented postnatal program for mothers. This intervention included a 1-hour training session before discharge from hospital which was followed by a phone call during the 2 weeks following discharge. Special IPT techniques were used, including providing information, using impact, transparency, marking for important cases, examining relationships and communication patterns, and providing social support. The control group received routine care. Outcome measurement was done prior to the intervention (pre) and at 6 weeks after delivery (post). According to the results, the level of social support in participants receiving IPT was significantly higher (Mean [SD] = 65.44 [8.43] versus 61.82 [9.99]; *P* = 0.009) compared with control group.^22^

####  Advanced practice nurse

 Hannan examined the effects of an APN telephone intervention for mothers in the postpartum period. The APN intervention included providing routine care in the hospital in addition to follow-up telephone calls to mothers during days 3, 7, 14, and 21, as well as 1 and 2 months after discharge. The APN spoke to the participating women on phone and inquired about whether they have concerns regarding their and the baby’s health. Outcomes were measured one day, three days, one month, and two months after discharge from hospital. The results showed that social support increased significantly in the APN group (Mean [SD] = 73.74 [7.95] versus 70.83 [10.61]) compared to the group receiving routine care 2 months after discharge.^28^

####  Self-help manual and a support group invitation

 Reid et al investigated the impact of two supportive interventions on postpartum mothers’ physical and mental health. In one intervention, postpartum women were invited to attend a support group dealing with postpartum held every week, starting two weeks after delivery. In the other intervention, postpartum women were sent a postpartum support manual (package) two weeks after delivery. Developed by the Maternity Alliance, the package offers supportive consultation to the mother (her health, what she needs in terms of sleeping and receiving support, how to deal with a crying baby, etc). The women attended these two-hour group sessions that were directed by facilitators in six central locations every week. In these sessions, the participants were asked to comment on issues related to their and the baby’s health. The findings were assessed 3 and 6 months after delivery, respectively. Finally, no significant differences were reported in postnatal social support between the control and experimental groups at all post-tests.^27^

####  Social support through the internet and at home

 Interventions providing social support based on the internet and at home were used in Sawyer et al. The study aimed to compare a group receiving a clinic-based postnatal health assessment as well as an internet-based group support, with a group receiving standard postnatal care support at home provided by a community nurse, during the 1-7 months after delivery. The internet support group was managed by a nurse who had received training on how to provide support to mothers through the internet. The groups were formed within the period in which the infants’ age ranged from approximately 1 month to 7 months. On average, each group included 12 mothers. Independent webpage designers had developed the website hosting the intervention. The design of the “chat” page in this study was similar to “chat rooms”, and an experienced obstetrics nurse provided and modified the content of this page. Nurses followed a curriculum that covered 11 broad subject areas related to infants (e.g., sleep, breastfeeding, and infant development) and mothers (e.g., maternal fatigue and breastfeeding techniques). A home-based support group received postnatal health assessment and pamphlets about care of babies in their home. At-home support visits by child and family health care nurses took 60-90 minutes. Outcomes were measured at baseline (4 weeks post birth), and 9, 15 and 21 months post birth. According to the results of the study, no significant difference was found between the internet-based group and the home-based group in terms of social support scores. Maternal outcomes in the internet-based support group were not inferior to those of the group receiving home-based postnatal support program. The internet was reported as an appropriate alternative to home-based support programs for the mother and the baby after delivery.^30^

 Jiao et al used web-based and home-based postnatal psycho-educational interventions. In the web-based group, mothers had exclusive access to a special website for a month. The web-based group was taught on how to work with the website. The home visiting group, on the other hand, received an intervention that was both home-based and psycho-educational which involved home visits and a pamphlet. Home visits were performed 5 to 10 days after delivery. Routine care was provided to the control group. Outcome measurements were done at baseline and 1, 3 and 6 months after delivery. Finally, at all post-tests, both interventions yielded better results in terms of social support compared with the control group.^23^ The length of each home visit was not mentioned.

 The study of Navidian et al, involved three sessions of intervention in which supportive-educational material was provided at home. Home visiting was done in the presence of husbands. Educational content addressed postpartum stressors, physical and psychological self-care, child care and social support. Outcome assessment was done following delivery at the end of the sixth week. According to the results, in comparison with the control group, lack of social support in the intervention group was significantly lower.^25^ The length of each home visit was not mentioned in this study.

###  Effectiveness of interventions

 The present systematic review aimed to examine the interventions made to improve maternal social support in the postpartum period. The effectiveness of interventions in improving social support was determined with higher scores of social support compared to controlled conditions. According to our results, six studies were found to be effective in improving the social support to postpartum mothers (as shown in Table 1 ). The following intervention types resulted in effective outcomes: (1) IPT,^2^ (2) APN,^28^ (3) home visiting,^23,30^ (4) internet support,^30,33^ and (5) counseling with men.^24^

 No significant difference between the intervention and control groups, however, was found in terms of social support in the remaining four studies which involved five interventions. These interventions were as follows: (1) a postnatal support manual,^27^ (2) invitation to attend a support group,^27^(3) CHW,^29^ (4) home visiting interventions,^26^ and (5) education package.^31^

 Interestingly, home-based interventions were both effective and non-effective in terms of improving social support to mothers during the postpartum period.

## Discussion

 The present study is the first systematic review that reports results of effective interventions for improving social support among postpartum mothers. To this aim, we reviewed 10 studies involving prenatal and postnatal interventions aimed at improving social support in postpartum mothers. The review covered nine interventions published in 10 studies of which 6 were found to be significantly effective in improving social support, whereas no significant results were found in the other four studies. In the six studies with effective outcomes, one prenatal intervention and four postpartum interventions were successful in improving social support as attested by the higher social support score of mothers in the intervention groups in comparison with the control groups. Of the interventions reviewed here, counseling with men which was performed in the prenatal period, had significant effectiveness in improving social support. Interventions performed in the postpartum period, including IPT, APN, internet support, and home visiting, also had significant effectiveness in improving social support. However, the intervention based on home visits showed both significant and non-significant outcomes in improving social support in comparison with controlled conditions. It can be argued that the social support of mothers is improved through certain mechanisms in these interventions. According to Barlow and Coren, for instance, educational programs for women along with their husbands improve maternal psychosocial health in various aspects of mental function.^33^ Heydari et al also found that in critical situations and stressful living situations, husbands play the most important role in providing support.^34^ Husband support contributes to promotion of physical and mental health and well-being of pregnant women and better birth outcomes.^35^ IPT is a psychotherapeutic approach that is manual-based.^36^ Evidence shows that IPT can be effective in developing postpartum psychiatric education programs for mothers.^37^ IPT deals with interpersonal relationships and emphasizes social interactions, communication, social support, and interpersonal performance. It has been shown that IPT intervention is also effective in promotion of social support, maternal competency, and reduction of depressive symptoms.^37-39^ APN’s use of telephone calls to track poor primiparous mothers in the postpartum period is a simple, reliable and low-cost way to enhance postpartum care, especially for those with problems, no health insurance, and difficulty to access the health care system, and those with financial and transportation problems.^40,41^ Alternatively, the internet can be used in order to overcome the limitations and shortcomings of face-to-face methods such as asking embarrassing questions.^42^ In general, the internet is an important tool for providing social support and removing social barriers since it makes access to information easy.^43^ A review reported that internet-based interventions significantly reduced depression in the postpartum period.^44^ During the postpartum period, home-based interventions as well as examinations of the mother’s and the baby’s health are of paramount importance. Such programs will promote the health of the mother and the baby, and improve their relationships with their husbands.

 Our review provides support for the advantages of the studied prenatal and postpartum interventions in improving social support among postpartum women.

 Regarding quality assessment, the quality of the studies was evaluated using a 7-item scale required by Cochrane guidelines. Most of the studies were reported as low risk, which is classified as good quality. Although the majority of the studies used an RCT design, many did not use blinding, and the reason was the nature of the intervention which left no possibility for blinding. Attrition bias was high risk only in three studies. Therefore, further studies with more methodological rigor are needed. Although our review showed that one prenatal intervention and four postpartum interventions had significant effectiveness in improving social support, there are a number of limitations in our study that are enumerated below.

 First of all, it was difficult to compare conclusions on the effectiveness of the interventions in each study, because this review involved studies that were different in terms of factors such as characteristics of participants, type of intervention, and tools. Therefore, we could not provide evidence for which intervention is most effective in improving social support among postpartum women. Moreover, we did not evaluate which intervention is the most cost-effective. Finally, the results of interventions reported to be effective in this review may not be generalizable to high-risk populations such as mothers with postpartum depression. Thus, future works are recommended to investigate how effective such interventions are in improving social support in high-risk mothers. Importantly, future studies need to focus on home-based interventions in improving social support in postpartum mothers, because such interventions have yielded mixed results in terms of their effectiveness in improving social support.

###  Research implications

 Despite the aforementioned limitations, this review is a unique project since there has been no similar review (to the best of our knowledge) on interventions aimed at promoting social support among postpartum women. The results of this study can pave the way for researchers and public health professionals for the introduction and development of interventions and strategies to promote women’s social support in the postpartum period. For future studies, interventions are suggested to evaluate the impact of the effective interventions reported in this study. In order to better promote social support among these women, it is also important to understand the factors that have an impact on their social support.

## Conclusion

 According to the results of this systematic review, interventions based on counseling with men in the prenatal period as well as those relying on IPT, APN, internet support, and home visiting in the postpartum period were successful in improving social support of postpartum women in comparison with controlled conditions. Interestingly, among these interventions, home visits showed mixed results in terms of their effects on improving social support. Therefore, prenatal care or postpartum care interventions can be tailored according to these effective interventions. However, based on the existing evidence, identifying the most effective intervention was not possible in this review.

## Acknowledgements

 The study was financially supported by Ahvaz Jundishapur University of Medical Sciences (AJUMS). The authors thank Mrs. Maryam Zahedian (head of the library of the School of Nursing and Midwifery, AJUMS), for helpful advice on the search strategy.

## Authors’ contributions

 FS: Project development, search and screening, quality assessment, interpretation of data, manuscript writing, approval of final version.

 ZA: Project development, search and screening, quality assessment, provided critical feedback, revision of manuscript, approval of final version. MJ: Project development, interpretation of data, provided critical feedback, revision of manuscript, approval of final version.

 ZBM: Project development, interpretation of data, revision of manuscript, provided critical feedback, revision of manuscript, approval of final version. MN: Project development, interpretation of data, revision of manuscript, approval of final version. BC: Project development, interpretation of data, revision of manuscript, approval of final versio.

## Funding

 This study was a part of PhD dissertation of the first author (Foruzan Sharifipour) which was financially supported by Ahvaz Jundishapur University of Medical Sciences in Iran (RHPRC-9919).

## Ethical approval

 This study was approved by the Ethics Committee of Ahvaz Jundishapur University of Medical Sciences (Ref. No.: IR.AJUMS.REC.1399.401).

## Competing interests

 The authors have no conflicts of interest to disclose.
